# Who Cares for Space Debris? Conflicting Logics of Security and Sustainability in Space Situational Awareness Practices

**DOI:** 10.1007/s11948-025-00550-3

**Published:** 2025-09-24

**Authors:** Nina Klimburg-Witjes, Kai Strycker, Vitali Braun

**Affiliations:** 1 Department of Science and Technology Studies, University of Vienna, Kolingasse 14-16, Vienna, 1090 Austria; 2 ESA Space Debris Office, Darmstadt, Germany

**Keywords:** Space debris, Security, Sustainability, Transdisciplinarity, Care

## Abstract

Satellites and space technologies enable global communication, navigation, and weather forecasting, and are vital for financial systems, disaster management, climate monitoring, military missions and many more. Yet, decades of spaceflight activities have left an ever-growing debris formation - rocket part, defunct satellites, and propellant residues and more - in Earth’s orbits. A congested outer space has now taken the shape of a haunting specter. Hurtling through space at incredibly high velocities, space debris has become a risk for active satellites and space infrastructures alike. This article offers a novel perspective on the security legacies and infrastructures of space debris mitigation and how these affect current and future space debris detection, knowledge production, and mitigation practices. Acknowledging that space debris is not just a technical challenge, but an ethico-political problem, we develop a transdisciplinary approach that links social science to aerospace engineering and practical insights and experiences from the European Space Agency´ (ESA) Space Debris Office. Specifically, we examine the role of secrecy and (mis)trust between international space agencies and how these complicate space situational awareness practices. Attending to the “mundane” practices of how space debris experts cope with uncertainty and security logics offers a crucial starting point to developing an ethical approach that prioritizes care and responsibility for innovation over ever more technological fixes to socio-political problems. Space debris encapsulates our historical and cultural value constellations, prompting us to reflect on sustainability and responsibility for Earth-Space systems in the future.

## Introduction

Decades of spaceflight activities have left an ever-growing formation of junk in Earth’s orbits—from rocket components, defunct satellites, and propellant residues to chips of paint, tools lost by astronauts, and fragments from continued collisions and explosions in space. Whether a byproduct of the Cold War era space race or the result of intentional destruction by spacefaring nations, space debris is an inherent feature of our ever-increasing activity in Earth’s orbital spheres (Clormann & Klimburg-Witjes, 2022; Damjanov, 2017; Klinger, 2021, 2019; Rand, 2019). Yet, space debris and its potential risks were not at all a concern during the first Space Age, which, as Rand (2019, p. 4) has argued, “became truly global not solely through acts of innovation but also in moments of decay.” Much like the plastics that give us microplastics or the production of nuclear power that gives us radioactive waste, space technologies were once heralded as innovations of modernity. However, as Reno has put it, “the invention of any substance is equally the invention of any of its accidental manifestations” (2020, p. 112) – demanding novel ways to mitigate the remnants of innovation. Further complicating the space debris problem, in what has often been called the new space race,^1^ has been the increasing commercialization and privatization of space. The proliferation of satellite constellations driven by companies like SpaceX and its Starlink program—while spurring innovation and expanding access to space-based service—has also dramatically increased the number of objects in orbit and has made the management of space debris even more challenging. With over 100,000 satellites planned for launch by private companies in this decade alone (cf. Langston & Taylor, 2024), it is clear that as orbital space becomes more crowded, space debris demands a fundamental rethinking of our ethical and political practices, and a consideration of proper intergenerational justice regarding the residues of our innovation activities (Felt, 2021).

This article began as an endeavor to understand the daily practices of space debris engineers at the Space Debris Office of the European Space Agency (ESA) in their ongoing quest to maintain active space infrastructure in the face of the ever-growing threat posed by space debris. However, in continued conversations among the article’s co-authors, a rather unanticipated theme began to emerge: the security legacies that are intimately entangled in the daily work realities of ESA space debris engineers. This paper is thus an investigation into these security legacies and the impact of this history on the present and future of space debris detection, knowledge generation, and mitigation and care practices. Specifically, we will attend to the multiple frictions, the role of secrecy, and the (mis)trust between international agencies that tends to hamper the global political action needed to sufficiently address the space debris challenge.

Acknowledging that space debris is not merely a technical problem, but a phenomenon that is deeply embedded in historical, ethico-political, and social contexts, we develop a transdisciplinary approach that links social science—STS and Critical Security Studies (CSS)—to aerospace engineering and practical insights and experiences from ESA’s Space Debris Office. Building on the STS tradition of “following” (cf. Latour, 1987; Latour & Woolgar, 1979; Akrich, 1992), we *follow with*, as a collective sense-making of complex scientific-technological practices, social science concepts, and aerospace engineering knowledge and practices. Our approach is reflected by a collaborative writing process comprised of continuous dialogues, iterations, conference presentations, and memo writing as well as co-teaching courses and presenting our work at interdisciplinary conferences. This article is thus not simply a synthesis of disciplinary perspectives or a form of “exploiting the experts engineer’s knowledge” (Passoth at al, 2021) but shows how interdisciplinary collaboration can yield new insights into complex, multifaceted issues like space debris. Such insights could challenge the dominant narrative of technological innovation as a panacea for socio-political problems (Pfotenhauer & Jasanoff, 2017; Haddad et al., 2024) and instead emphasize the importance of integrating questions of care, maintenance, and ethical responsibility into the governance of innovation and its leftovers (cf. Felt, 2021).

The first section of this article introduces space debris as a growing challenge to orbital sustainability, foregrounding its multiple security-related legacies. Following this, the second section outlines our conceptual approach. Rather than observing from the outside looking in, we instead engage in a mutual exchange of perspectives and questions about care and responsibility for space debris. This exchange is carried out in the form of an ongoing conversation between social scientists and aerospace engineers as an experiment in collaboration (Passoth et al., 2021) to capture the struggle to manage the “lively, energetic and sometimes unruly behavior of materials and objects” (Walters, 2014, p. 102) from a transdisciplinary view. These conversations—semi-structured, recorded, transcribed, and collectively reflected on—blend scientific practices, social theory, and engineering knowledge. While not an ethnography in the stricter sense, our approach builds on examples of collaborative ethnography, with an intricate—albeit occasionally clumsy—dance between researchers and their subjects to craft ethnographic narratives (see Lassiter, 2005; Boyer et al., 2015) together.

The empirical section is divided thematically into three parts to discuss: material choreographies, the technopolitical legacies of space situational awareness infrastructures, and the complex relations between techno-epistemic uncertainties, security, and secrecy. Each part begins with a vignette from the daily work practices at the ESA Space Debris office. The conclusion sketches out a larger critique of the dominant discourse that more technological innovation is the solution to the space debris problem. We argue that we must instead rethink our ethical responsibilities towards the shared outer space commons.

## Space Debris, a Security Legacy

On January 11th, 2007, a ballistic missile was launched from the Xichang Space Launch Center in the southwestern Chinese province of Sichuan. It ascended 863 km above the Earth’s surface and met the defunct weather satellite Fengyun-1 C head-on (Weeden, 2010), obliterating it completely and scattering a cloud of more than 3,000 fragments into the orbital environment, causing “the worst contamination of low Earth orbit in history, creating a debris pollution that will last for decades” (Anz-Meador et al., 2022, p. 456).

16 years later, in August 2023, following a “Predetermined Debris Avoidance Maneuver,” the International Space Station (ISS) fired its thrusters to avoid a possible conjunction—a term used to describe when two objects in orbit are presumed to experience a close approach—with a piece of debris from the 2007 Chinese anti-satellite test (Cowardin, 2023). Such avoidance maneuvers are increasingly common for space stations and active satellites alike, which must protect space assets from tens of thousands of cataloged pieces of space debris that continuously circle Earth. The 2007 anti-satellite (ASAT) test is a stark reminder of the long-lasting impact such events can have on the orbital environment. The debris from this event alone continues to pose significant risks for active satellites and space stations, highlighting the sociotechnical vulnerability (Hommels et al., 2014) of our space infrastructures—infrastructures our modern societies rely on to support everything from communication and navigation to military operations and scientific research. A disruption of any of these services would not only create critical security issues, it would also be economically costly.

Space debris is different than other remnants of modernist technological ambitions: its breakdown renders its disposal—if not impossible—highly difficult. When a satellite becomes unresponsive, it instantly becomes space debris; it will haunt whatever orbit it is on for months, years, decades, or centuries, sometimes even millennia to come until it is removed or burns up in the atmosphere (ESA, 2023, p. 64). This reversal is somewhat ironic. What had previously been an expensive asset worthy of costly protection from space debris becomes the very thing it was previously protected from: a high-speed projectile uncontrollably tumbling its way through space, endangering everything that is and everything that will stand in its path (Clormann & Klimburg-Witjes, 2021). A material legacy of past spaceflight aspirations and a threat to current and future aspirations all at the same time, space debris poses interesting questions about the temporalities of innovation in technology-driven societies, especially with regard to security concerns, military logics, and infrastructural geopolitics (cf. de Goede & Westermeier, 2022).

During the first space race- from the launch of the first satellite in 1957 to the end of the Cold War in 1989, about 70% of satellites launched served military purposes (Harrison et al., 2017). There are many indicators that suggest that significant portions of the space debris population originate from carrying out security logics. Predecessors of today’s ASAT tests, debris from Soviet-era co-orbital ASAT tests are still, more than a half-century later, in orbit (Weeden, 2022). The need to maintain secrecy has long been at the forefront of the reasoning to destroy these satellites with explosive charges that scatter these fragments into the orbital environment, some of which remain to this day, even decades later.^2^ As the NASA report on fragmentation events in orbit points out: “Deliberate actions, often associated with activities related to the launching state’s national security interests and concerns, were formerly the most frequently occurring class [of space debris generating events]” (Anz-Meador et al., 2022, p. 28). Following the peak of such fragmentation events in the 1970s and 1980s came an increase in research about the possible consequences of pollution on Earth’s orbital spheres. This research is perhaps most prominently exemplified by Donald Kessler’s concerning prognosis that a chain reaction of self-propagating in-orbit collisions could make some orbital regions practically unusable for generations to come and significantly obstruct astronomical observation (Kessler & Cour-Palais, 1978). Given this historical context, it is clear that the remnants of past military activities continue to influence both current and future states of Earth’s orbital environment. We thus understand space debris to be material residues of security regimes, enduring to this day and continuing to affect outer space futures in a variety of ways.

However, as we show in this article, not only did space debris originate from military missions and security logics that have always been closely entangled with geopolitical tensions but, to this day, our knowledge about space debris has been intimately enmeshed with security rationales. This consequently creates particular forms of non-knowledge and strategic ignorance (cf. Oreskes, 2021; Aradau & Perret, 2022) that continue to obstruct any chance for a collaborative and global approach to address the space debris issue.

## State of the Art

Humans have transformed Earth’s environments since the beginning of space flight. Yet, only recently have the social sciences focused on questions of sustainability and responsibility for outer space environments (Klinger, 2019). Outer space, despite “being often imagined as an empty vessel (…) awaiting purposeful inscription by the human species” (Kearnes & van Dooren, 2017, p. 182) is deeply affected by technopolitical endeavors beyond the stratosphere. Attending to the interplanetary space as a “cultural landscape forged by the organic interaction of the space environment and human material culture,” as Gorman (2005, p. 86) has suggested, allows us to consider junk in space not as a distant outer space phenomenon but rather, in many ways, to be closely bound to planetary concerns (Hunter & Nelson, 2021, p. 229; Praet & Salazar, 2017). Recent years have seen a growing interest in the topic of space debris in several different fields. There has been, for example, a focus on the need for more polycentric governance of space debris (Morin & Richard, 2021), on space junk as material cultural heritage (Gorman, 2020), on discursive strategies in the European Space sector to link space sustainability to terrestrial sustainability problems (Clormann & Klimburg-Witjes, 2021; Yap & Truffer, 2022) and environmental (in)justice (Klinger, 2019; Marino & Cheney, 2023), legal issues (Kopal et al., 2011), and suggestions for novel forms of international regulation (Migaud, 2020). As Damjanov has put it:

While our governance of waste in orbit is grounded in political and economic rationalities and constrained by our techno-scientific capacities, also needed are instruments of policy and planning that touch upon things that are deeply human. The matter of orbital debris demands fundamental developments in ideas about politico-ethical practices, the life and death of technologies, and the status of what is shared. (2017, p. 180)

Particularly illuminating in this light has been Yap and Kim’s (2023) and Yap and Truffer’s (2022) work on “Earth–space sustainability,” which highlights the growing interconnection between sustainability challenges on Earth and those in outer space and argues that they are now too intertwined to be treated separately. They emphasize that, on one hand, activities in Earth’s orbits—such as satellite deployments and space exploration—directly impact environmental and social sustainability on Earth. On the other hand, Earth-based environmental policies influence space activities, particularly as the demand for orbital resources and services grows. This analysis allows us to explore the distinction between sustainability *through* Earth’s orbits and sustainability *in* Earth’s orbits. While the former refers to how orbital technologies like Earth observation satellites can contribute to solving sustainability issues on the ground, the latter addresses the need to manage growing environmental pressures within Earth’s orbits, such as space debris and orbital congestion, to ensure the long-term viability of space as a resource for future generations (Klinger, 2021; Marino, 2023).

Recent work on Space Situational Awareness (SSA) and Space Traffic Management (STM) has been crucial in addressing the growing sustainability challenges in near-Earth space. SSA focuses on monitoring and modeling space debris and active objects in orbit to provide data crucial to understanding the risks posed by increasing orbital congestion. STM, on the other hand, encompasses the regulatory and technical frameworks necessary for managing the safe and efficient use of space to ensure that space activities remain viable in the long term. Oltrogge and Cooper (2020) have highlighted the gap between current SSA capabilities and the ambitious goals of STM, which aim to not only track space debris but to actively manage space traffic to prevent collisions and preserve orbital space. Meanwhile, Ailor (2015) has underscored the importance of international cooperation and regulatory coordination for effective STM, emphasizing that without robust traffic management, the risks of collisions and space debris accumulation could undermine the sustainability of space activities. Together, these works provide a critical foundation for understanding the complexities and challenges of maintaining sustainable operations in increasingly crowded orbits. This paper aims to add to these relevant studies with a perspective that attends to the daily practices of space debris engineers to investigate how infrastructures for tracking and mitigating space debris can be found in the mundane and seemingly boring arenas of sociotechnical relations (Leander, 2021, p. 163; Huysmans, 2011) while so closely entangled with global security regimes that have been extended towards outer space.

Much work on infrastructures in STS has drawn attention to the silent (or silenced), laborious, and often controversial practices, which enable infrastructural operations, mechanisms, and effects—including monitoring activities, standardization procedures, and repair or maintenance interventions. Even if infrastructure tends to disappear into the background of social life, its decay and moments of breakdown can serve as revelatory spectacles. Here, infrastructure is cast into the sharp view of all participants and an acute moment of visibility ensues. Rather than staying hidden as its prefix suggests, infrastructure becomes most noticeable as it breaks down (Bowker & Star, 1999; Larkin, 2013). While designers of infrastructure often imagine their new creations to be adaptable to future challenges, to be suitable for any context, these systems are ultimately shaped by specific historical and local conditions; this makes them susceptible to unforeseen changes (Edwards et al., 2009, p. 371). In other words, while infrastructure aims to last well into the future, its longevity is never guaranteed.

Bowker and Star’s often cited methodological plea for infrastructural inversion exemplifies this analytical strategy: to look into the arrangements that “tend to fade into the woodwork” and recognize “the depths of the interdependence of technical networks and standards, on the one hand, and the real work of politics and knowledge production on the other” (1999, p. 34, cf. Star & Ruhleder, 1996; Edwards, 2002; Graham & Thrift, 2007).

To better understand the complex entanglements of infrastructures for debris mitigation, space surveillance, and tracking with security logics and military legacies, we must look more closely into what Star (1999) has alluded to as the **“**boring things,” which nonetheless secure our experiences of everyday life and order. In the case of space debris, this also speaks to what Jackson (2014) has called “broken world thinking,” a research perspective that is empirically and conceptually centered around “erosion, breakdown, and decay, rather than novelty, growth, and progress” (p. 221).

Here, work in STS on the notion of care offers an important lens through which to understand the social, political, and ethical dimensions of technoscientific practice. Care, in this tradition, is not only an affective or moral disposition but a material, relational, and often contested practice that involves attending to the needs of humans and nonhumans, technologies, infrastructures, and environments. Foundational contributions such as Fisher and Tronto’s (1990) feminist theory of care have emphasized the deeply political nature of caring labor, while more recent STS scholarship has traced how care emerges in the practices of scientists, engineers, and policymakers who navigate complex sociotechnical worlds (Martin et al., 2015; Mol et al., 2010; Puig de la Bellacasa, 2011). Davies (2019) has explored how scientists enact responsibility and care in their everyday work, especially in contexts where neoliberal pressures and public accountability regimes increasingly shape science. Care, as she argues, is entangled with questions about living ethically within institutions that do not always reward slowness, reflection, or hesitation.

This body of work conceptualizes care as a practice of “tinkering” (Mol et al., 2010), a situated and often improvised engagement with messy realities, and as a mode of attention that involves translating between different epistemic and normative commitments (Coopmans & McNamara, 2020). Care is also understood as deeply entangled with power, as decisions about what or who is cared for—and how—are always shaped by broader political and institutional arrangements (Gill et al., 2017). These insights have encouraged scholars to pay close attention to the everyday work of maintaining, adjusting, and responding within technoscientific systems—not as secondary to innovation, but as central to how knowledge and technologies are made to work in the world (Latimer, 2000; Law, 2010; Lydahl, 2021).

We draw on this rich scholarship to explore how care is practiced and hindered in the context of space debris mitigation, which is increasingly becoming a matter of care an object around which new forms of ethical attention and responsibility are coalescing (Puig de la Bellacasa, 2011).

Through interviews, recorded and transcribed conversations and seven co-writing sessions, joint conference presentations and shared classroom encounters with students from various disciplinary backgrounds^3^, we examine questions of accountability, care and repair in space debris mitigation work. Space debris engineer`s practices of care may not always appear as such; they are often embedded in risk assessments, standard setting attempts and their political complications (Bowker & Star) or operational routines. Yet, as Pols (2008) and Martin et al. (2015) remind us, care in technoscience is frequently subtle and distributed, emerging in efforts to make technologies more responsive to the fragilities they encounter or produce. In turning to maintenance as care, we aim to foreground these seemingly mundane yet consequential practices within the broader political economy of space innovation—a context where doing less, slowing down, or maintaining what already exists may be more ethically significant than pushing for the next breakthrough.

Building on these perspectives, our article thus aims to contribute to a growing body of work that is interested in the practices of care that maintain, repair, and adapt our complex socio-technical world and make life as we know it possible (Damjanov, 2023; Ramakrishnan et al., 2021; Jackson, 2014; Graham & Thrift, 2007).

### Conceptual Approach

Taking the material agency of residues seriously thus calls for attending to people and practices. Empirically, this means following space debris engineers and their daily struggle to tame the risks of space debris. Such approaches have already been taken up in Critical Security Studies with work that attends to the practices that inform and produce in/security by studying everyday procedures and routines (Cornut, 2015; Hönke & Müller, 2012; Côté-Boucher et al., 2014). Thinking with Acuto (2014), the “everyday” of space debris engineers can be understood as situated, mundane, and habitual practices of global governance. For instance, in the context of space debris management, political processes—international, national, and local—must not only be executed, they must also be translated into a routine reality. In the STS tradition of “following”—tracing the development and transformation of scientific and technological objects, practices, and actors to provide a dynamic and relational perspective on knowledge production and technology development—we will *follow with* by attending to the space debris engineers’ everyday work practices.

In STS, the notion and practice of “following” is rooted in Actor-Network Theory (ANT), a body of thought that posits that scientific and technological outcomes are the result of networks of human and non-human actors alike. Within an ANT framework, actors continuously negotiate and redefine their roles, relationships, and boundaries. These mostly ethnomethodological approaches (Garfinkel, 1984) emphasize the importance of understanding the everyday practices and interactions that constitute the socio-technical reality, “following” scientists and technologists in their daily work and observing how they create, interpret, and sustain their activities and highlighting the co-constitutive relationship between technology and society (Latour & Woolgar, 1979; Akrich, 1992; Knorr-Cetina, 1999; Mol, 2002). Subsumed under the label “laboratory studies,” these and subsequent works have demonstrated how laboratory instruments, experimental protocols, and the social dynamics among scientists help stabilize scientific knowledge. Still, although the “following” approach has been productive, it also tends, at times, to be overly descriptive, focusing on micro-level practices at the expense of broader structural and political dimensions.

In this paper, we thus attempt to follow slightly differently as the social science authors do not ‘come to the lab’; instead, we follow with *follow with* through a mutual exchange of perspectives and questions related to forms of care and responsibility for the material legacies of innovation.

We agree with Liden and Lydahl (2021) that the methods we use shape how care can be analysed and practiced. Drawing on Haraway (1997), methods can be seen as “wonderfully detailed, active, partial ways of organizing worlds” (p. 90), underscoring their role in producing situated versions of care. Ethnographic storytelling, as illustrated by Mann and Yuille (see also Winthereik & Verran, 2012), demonstrates how ethnography can hold together the tensions between everyday practices of care and broader policy ideals—making visible alternative enactments of care that might otherwise be overlooked (cf. Moser, 2011; Martin et al., 2015).

Written as a continued and iterative conversation between researchers from social science and aerospace engineering, this paper is an experiment in collaboration (Passoth et al., 2021); it aims to capture the struggle to tame the “lively, energetic and sometimes unruly behavior of materials and objects” (Walters, 2014) from a transdisciplinary perspective. The “conversations” and vignettes that follow are the result of various forms of semi-structured exchanges and iterative reflections, recorded, transcribed, and commented on by all of us. They are a collective sense-making of complex scientific-technological practices, theoretical social science concepts, and aerospace engineering knowledge and practices.

This method of writing together can at times resemble a clumsy dance. It is not always fluent or symmetrical—there are missteps, moments of confusion, and interpretative gaps where we do not fully understand what the other means. Yet it is precisely in these moments that care becomes most palpable. As Puig de la Bellacasa (2017) reminds us, care is not only about affection or ethical intent but also about the labor and commitment required to engage with others’ concerns—even, and especially, when they are not immediately legible. Writing together thus becomes a situated practice of care-fulengagement, where the act of translating technical, disciplinary, or theoretical knowledge for each other is itself a form of companionship. Linden and Lydal (2021) similarly suggest that care in transdisciplinary settings often emerges through acts of maintenance, coordination, and sustained attentiveness to difference. Martin et al. (2015) argue, care in STS is often enacted through “doing things together,” in which knowing, tinkering, and responding to one another’s intellectual and affective investments become central to how research gets done. Our writing, then, is both epistemic and affective. It is a shared mode of inquiry that proceeds not from consensus, but from an ongoing responsiveness to each other’s methods, terminologies, and hesitations. It is in this awkward, improvisational process that we cultivate the possibility of staying with complexity rather than reducing it.

## Empirical Section

In the next three sections, we introduce and discuss the broader socio-political context that shapes the work of space debris engineers and how security concerns come to shape orbital care work. Each section begins with an anecdote from the life of our space debris engineer co-author. This is intended to empirically center our work around the grievances of space infrastructure care workers, allowing us to highlight the underlying issues related to the ever-present security regimes.

### Material Choreographies and Epistemic Uncertainty: Taming Infrastructural Threats in an Unpredictable Domain

It was one of the usual Tuesday nights when the members of the Space Debris Chess Club—enthusiasts of the game of chess and typically close to the work in the domain of space debris—would meet online again for a joyful time. I was on call that day and a warning came in, with the collision probability above our threshold, and our reaction time being only about 10 h. I left the meeting to initiate the first additional screenings and study maneuver options for the Argentinean Earth observation satellite we are supporting. In parallel, I reached out to their flight control team via phone. Probably a hiccup of the transatlantic connection, which did not allow me to get to talk to them, but fortunately, the reaction came in by email already. The maneuver had to be prepared and uplinked by about 3 a.m. More work, less chess, less joy, less sleep. Only 3 to 4 h that night, as the next business day would start at regular time again.

Dozens of these collision warnings are received by space operators each day and the alerts are expected to grow rapidly in the coming years (Yap & Kim, 2023; Greenbaum, 2020; Krag, 2021). Striking in the anecdote above is just how uncertain and ill-timed the work reality of space debris engineers is at the European Space Operations Center, how the volatility of space debris—or even the volatility of available information about space debris—affects their work practices. The erratic collision warnings that cause the engineers’ night-time disruptions, however, arrive following a complex material interplay—between debris in space and the earth-based instrument assemblage that monitors orbital objects, both active and abandoned. On Earth, the space debris sensing regime is primarily empowered by two different methods, each of which is at the base of our collective understanding of space debris: optical telescopes and radars. Because these two types of instruments intra-act with the materiality of space debris quite differently, they thus enact space debris differently (Mol, 2002; Barad, 2003).

Optical telescopes, much like binoculars, suffer from a key limitation: a narrow field of view. This limitation becomes critical when tracking fast-moving objects, which often appear in images as mere smudges and require elaborate analysis to determine their orbits. In addition, while telescopes can track objects beyond radar range, they also depend on external light sources like the sun. This restricts their effectiveness, meaning that they can only viably be used at night and that they are vulnerable to certain weather conditions such as cloud cover. The effectiveness of telescopes is also influenced by the material properties of the satellites: their size, shape, and reflectivity.

In contrast to telescopes, radars use electromagnetic waves to detect orbital objects; similar to sonar, this allows them to measure distance, direction, and velocity. However, this range is limited, so radars are typically only used to track objects in low Earth orbit (LEO). The radar’s effectiveness is dependent on the material composition, size, and shape of the object—while some materials are highly reflective to radar waves, others are nearly invisible. As a consequence, objects smaller than 10 cm in LEO and anywhere from 30 cm to 1 m in geostationary orbits are often undetectable by radar. This leaves a significant gap when tracking smaller debris, making their estimated number largely speculative and offering limited practical value to the spacecraft operators who protect space infrastructure.

Recounting how space debris comes to be enacted multiply is not simply an effort to further reify the conceptual body of Mol (2002) and others nor is it an attempt to squeeze in yet another example of how matter comes into being through situated practice. Instead, it is aimed to highlight and demonstrate the temporal and spatial complexities of the material agent that is space debris and how this agent intersects with the lives of people on Earth. The problem of space debris itself is inextricably linked to its materiality. But this materiality must also be epistemically translated into the social world: it becomes visible and actionable through instrumental assemblages, which are employed to track the debris on their way through orbital spheres.

To make the collected data useful, it thus needs to be extrapolated into the future. The position, direction, and speed of each object are used to update a model of the space environment, which predicts where each object will be over the next 7 days. However, all measurements come with uncertainties. To account for these, objects in space are computationally represented as ellipsoids: stretched bubbles that reflect the uncertainties in their position. Since space objects move at very high speeds (over 25,000 km/h in LEO), the biggest errors are usually in the direction of travel, elongating the ellipsoids along that axis. If these ellipsoids overlap along their predicted paths, an alert message, or “conjunction data message,” is generated and sent to spacecraft operators.

As described in the anecdote above, combined with irregular tracking updates of the instrumental assemblage, epistemic uncertainties^4^ (cf. Knorr-Cetina, 1999) about the position, direction, and velocity of objects in space fundamentally shape the particular work temporalities of space debris engineers. In these instances, the uncertain and fuzzy intra-action between orbital objects and tracking infrastructure becomes visible, and demands immediate action from the responsible spacecraft operators to ensure the continued safe operation of space infrastructures. Thus, with the influence of the fluctuating space weather, epistemic uncertainties are the primary reason for the ESA’s level of organizational preparedness. In other words, epistemic uncertainties structure the way in which the labor of space debris engineers is organized. Within the operational sphere of the ESA Space Debris Office (SDO), a group of specialized engineers engages in a rotational duty system, whereby each individual assumes a 24/7 on-call status for a week at a time. This duty entails that the engineer always carries an on-call phone to promptly respond to incoming collision warnings. When they do receive such notifications, the engineer must mobilize swiftly; in some cases, they must travel immediately to the office premises of the European Space Operations Centre (ESOC) to plan and execute collision avoidance maneuvers.

Knowledge about space debris, however, is not only often uncertain due to space debris material characteristics and agency, but also because of how intimately it is enmeshed with security rationales, which continues to create particular forms of non-knowledge (cf. Oreskes, 2021; Aradau & Perret, 2022) that obstruct a collaborative and global approach to addressing the space debris issue. This is encapsulated by the vignette below.

### Technopolitical Legacies of Space Awareness Infrastructures

“Excuse me, sir—are you speaking in French?” the U.S. soldier on the other end of the line was asking me. It was about 2 a.m. in the morning on my side and his call had just woken me up. According to procedure, he would notify me via phone about a conjunction event that had crossed a collision probability threshold of 1%. “I just said that I understood what you were saying and that we had already anticipated this event and prepared an evasive action,” I replied to him. My English may be far from perfect but must have intertwined in that moment with a noisy transatlantic phone connection and my body trying to express language after being pulled out from nightly dreams within an instant—to basically strip our conversation of its purpose. “Sir, I really have trouble understanding you. But I hope you understood me. Have a nice day.” And that ended our call.

For the uninitiated reader, it might seem surprising that a European space debris engineer is rudely awoken by an American soldier in the middle of the night. What does the American military have to do with the safe operation of European space assets? The historical development of American Space Situational Awareness infrastructure and data processing is a story inherently linked to the expansive reach of American power, well beyond its own territory and on into space. It is exactly this reach that makes American space situational data superior, and thus more useful, for commercial and European satellite operators. Today, the American Space Surveillance Network (SSN), the name for the web of radar installations, optical telescopes, and dedicated satellites operated by the U.S. Space Force and related security actors, spans the entire globe, from the farthest reaches of Alaska to remote islands in both the Pacific and Atlantic.

Space debris is a material legacy from previous decades of spaceflight—so too are the infrastructures to monitor, measure, and detect it. In addition, these infrastructures, be they material or institutional, are generally located within national security apparatuses. Efforts to mitigate space debris thus often rely on infrastructures that were built for very different purposes—usually under the auspices of defense departments and national security agencies. This cannot be overlooked. As Star and Bowker have contended, in relation to infrastructures, “[e]ach subsystem inherits, increasingly as it scales up, the inertia of the installed base of systems that have come before” (1999, p. 33). Thus, when it comes to space debris, security interests continue to shape the production and circulation of data, which is collected by infrastructural projects that are financed, planned, constructed, and operated by military or other national security actors, such as intelligence services (cf. Oreskes, 2021). The infrastructure for space debris tracking may serve as an exemplary instance to explore the influence and consequences of military patronage in large infrastructural projects (Oreskes, 2014). This infrastructural legacy becomes evident when following with the daily work of space debris engineers who rely on a space debris catalog that has been maintained and kept up to date by the U.S. Space Force and its institutional predecessors, which were also thoroughly integrated within the U.S. military apparatus. This integration is not an America-only phenomenon. In Europe, space surveillance infrastructures are often controlled and operated by military actors as well and they too are deeply integrated into the European security apparatus (Peldszus & Faucher, 2022). Today, even if private companies did begin to build dedicated infrastructure of their own, it would be tough for them to rival the current space debris catalog, which was first established in 1961 and remains the industry standard for space debris tracking.

The social and material legacies of the tracking infrastructure shape the knowledge that is being produced about space debris. However, what it equally constitutes are instances of non-knowledge: epistemic blind spots that can be explained by the particular form the infrastructure has taken during its historical development. Even if the American Space Situational Awareness infrastructures provide spacecraft operators collision warnings today, U.S. radar infrastructures were initially built to be early warning systems that scan the sky in search of Intercontinental Ballistic Missiles (ICBM) armed with nuclear warheads. Tracking capabilities that could distinguish what is and what is not space junk were thus developed with this primary imperative. For example, radar stations to track objects in LEO were among the most prominent to have been built during the Cold War and they were designed to identify and track Soviet ICBMs on their way through space. The use of these radar installations for the surveillance of space has thus never been their primary mission. In fact, there is only one U.S. radar installation^5^ that is fully dedicated to the surveillance of space objects, and this is only because other radar installations took over its ballistic missile warning function in 1987 (cf. Chatters & Crothers, 2009). Even though these radar and optical telescope stations are scattered around the world, they are particularly concentrated in the north-western hemisphere (Geul et al., 2017), as they served and continue to serve the purpose of protecting North America from the threat of nuclear annihilation by geopolitical rivals. Because America’s geopolitical rivals have also been located in the northern hemisphere, there is a considerable lack of tracking infrastructure in the southern hemisphere. This particular setup, born from Cold War era national security priorities, has also led to blind spots in the coverage of space debris (cf. Johnson et al., 2008).

This leads us to conclude that what we know and, equally as crucial, what we do not know about space debris is structured through a technological assemblage that grew out of Cold War fears of nuclear war. This resonates with Howe and colleagues (2015) assertion that: “A lesson of infrastructure is that it surfaces the social conditions and times in which it is sited; thus, it demonstrates as much about our historical and cultural attentions in a particular moment and place as it does about the thing itself.”

Still, it is not only the sensor network that is under the control of the U.S. Armed Forces; the U.S. Space Force also maintains the data infrastructure used to evaluate collected data and the space object catalog. This securitization of data and processing capabilities structures the work of space debris engineers at ESA considerably, who, as exemplified by the anecdote above, must ultimately rely on the U.S. Space Force to evaluate evasive trajectories and ensure that the paths chosen for satellites are safe. In this sense, the data infrastructures of the space debris catalog—which tracks the formation, spread, and multiplication of waste—are themselves a security legacy of the Cold War competition for power in space.

### Orbital Opacity: Security Regimes and Secrecy as Obstacles to Care

There is usually enough time to prepare a collision avoidance maneuver. On that day, I escalated to the flight control team an event with about three days lead time to the event, in line with our procedure. The secondary (or chaser) object appeared to be a satellite, which means that we would have to find out whether it is still operational and, if so, who operates it. And that has to happen rather quickly, as coordination requires time. I saw that the other satellite has been in orbit only for a couple of years, which makes it likely that it would still be operational and not an abandoned piece of hardware. There is no comprehensive database or dedicated entity keeping track of the current activity status of all spacecraft. At space-track.org, where operators may voluntarily provide contact information and update their satellite’s activity status, there was no entry for the Earth observation satellite, which appeared to fall under jurisdiction of the Russian Ministry of Defense. I was able to find their website, but there was only very limited contact information. A generic ‘info@’ email address was all I could work with. With the consensus of our team, we informed via that channel about the situation and what we are intending to do, asking to forward that email to the responsible flight control teams of the other side. The days passed and we did not get a reply. We decided to proceed and sent another email essentially saying, “Whoever reads this—we are maneuvering, please stay put.”

The preceding vignettes should have made clear that space debris is the link between very different communities of practices. On one hand, there are civil satellite operators whose primary goal is to ensure space safety; on the other hand, there are military actors whose duty is to fulfill the ends of military security logics. Being the link between these two spheres, space debris can thus be understood as a “boundary object^6^” (cf. Star & Griesemer, 1989). Beyond this linkage point, however, the intersections and interactions between these different regimes and logics often bring about a host of frictions and complications. A particularly routine cause for such difficulties is the imperative for secrecy common within the security domain (cf. de Goede et al., 2019; Klimburg-Witjes et al., 2022; Gusterson, 2008). This imperative for secrecy is exemplified by the outright omission of satellites in public catalogs when they are deemed relevant to security. The fact that most space surveillance infrastructure is controlled and operated by national security actors only compounds the uncertainties about how tracking data is produced. Beyond hindering interoperability and making comparisons between different data providers cumbersome, if not practically impossible, these uncertainties also reify the reliance on American information and infrastructure.

The military patronage of the space surveillance sector is likely to persist. Current ground-based tracking infrastructure is susceptible to weather and other atmospheric disturbances, and national security actors have increasingly favored space-based capabilities. For example, in a recent project called “SilentBarker,” the United States Space Force, in collaboration with the U.S. National Reconnaissance Office, deployed space reconnaissance satellites. However, due to the secrecy of the project, very little is known about their actual capabilities. Yet, some statements have alluded to a focus on geosynchronous orbits, which, to date, have been outside the reach of most Earth-based sensors because of how far removed they are from the Earth’s surface (Tingley, 2023). Developments such as these make clear that state-of-the-art space surveillance data continues to be controlled by military or national security actors and will likely continue to be unavailable to civil and scientific stakeholders (cf. Witjes & Olbrich, 2017).

Furthermore, secrecy also has a significant impact on the daily working conditions of space debris engineers, particularly as the sensitive nature of the data they are privy to makes them subject to heightened securitization processes. As Braun experienced daily in his work at the Space Debris Office, these growing security measures—including more stringent security clearances, the establishment of dedicated security zones, and the implementation of restrictive security regulations—further complicate the operational environment for spacecraft operators and create yet more barriers to openly addressing space debris as a public concern. This dynamic is particularly evident when security protocols are introduced that strictly regulate interactions with citizens of high-risk countries; ostensibly carried-out to prevent industrial espionage, they can often effectively stigmatize entire populations. Spacecraft operators and engineers also face increased administrative burdens, such as the requirement to clear or replace their electronic devices before they travel to these high-risk countries to prevent the inadvertent transfer of classified information.

Beyond secrecy, the organizational structure of military institutions—characterized by rigid bureaucratic and hierarchical systems—only exacerbates these challenges further by hindering opportunities for spontaneous and flexible problem-solving, and limiting informal communication channels. This bureaucratic rigidity within the space security regime generates a persistent opaqueness that complicates the safe operation of space assets by civilian operators, as is strikingly illustrated in the vignette above.

Still, secrecy and the inherently hierarchical structures implicated security actors operate in are not the only causes of the many challenges European space debris engineers experience. The next vignette illustrates the complex entanglements of different uncertainties that adversely impact the care and maintenance work of space infrastructures. It demonstrates how space debris becomes a “wicked problem” that resists any straightforward resolution.

## Discussion: The Co-Constitution of Techno-Epistemic Uncertainties, Security Regimes and Secrecy

The debris piece we monitored for a few days showed rather volatile behavior: the collision probability would go up and down, crossing our reaction threshold several times. This was mostly because it is a small object, which is difficult to track, especially during phases of high solar and geomagnetic activity. We started preparing a collision avoidance maneuver and kept monitoring any potential updates we would receive in parallel. The nature of such objects has it that it’s also very difficult to know if and when new information may arrive. Even if we ask the operators at 18th Space Control Squadron to increase tracking or learn more about their intentions to improve orbit information on the object of interest—we may not get any really actionable feedback. That day was one of those, where we would have to live with that uncertainty. Moreover, the planned avoidance maneuver required screening, to make sure we would not be flying blindly on a new path and into any new potentially high-risk events. We were on time and had anticipated the 2–4 h turn-around to get that feedback for our new orbit. Except that it never arrived. But that day there was no more margin left. After having waited for about 5 h, we had reached the decision point, and the mission manager had to decide: do you trust the available information on this volatile debris piece? Does it represent the risk we assess, given that we had not seen any updates in quite a while? Does it warrant conducting a collision avoidance maneuver given that the latter had not received any screening? How do you weigh one risk against the other in such a situation? In this instance, it was decided to execute the maneuver.

In this vignette, the different difficulties of space debris that we analytically differentiated in previous sections come together in a way that exemplifies how their co-constitution creates a complicated conundrum for the care workers of space infrastructures. What might be analytically separable acts jointly in practice. Each of the difficulties exist simultaneously and are impossible to disentangle in the enfolding process.

The opaque practices of data gathering for space surveillance, the uncertain influences of the space environment, and the structural limitations in communication between civil spacecraft operator and military space surveillance professionals—due to secrecy and the material agency of space debris itself—remind us of Walters’ notion of ghostly objects, which “can be fleeting, ambiguous, partial, absent-present, ghostly and more-than-single” (2014, p. 103). Secrecy, as Walters writes, gives rise to situations where the visibility and knowability of objects is far from straightforward (2014, p. 103).

Ultimately, these complex interactions lead to ambiguous and ghostly space debris objects that are not to be tamed easily. When combined with the epistemic uncertainties discussed previously, the opaqueness of secrecy makes space debris a somewhat spectral apparition with its own “unruly behavior” (Walters, 2014, p. 102), setting time frames of but a few hours for engineers to work with, which can be impossible to reach. For ESA satellites in LEO, conjunction messages contain information about projected events up to 7 days in advance so that critical events can be prepared for and responded to. Monitoring updates regularly, first collision avoidance meetings typically begin 3 days in advance to prepare for evasive maneuvers. Given the volatile and unpredictable nature of how space debris is known to space debris engineers, it is a rather common occurrence that these planned maneuvers are never actually executed. In fact, about three-quarters of these maneuvers simply become obsolete. Still, this is always a what-if scenario and work, time, and resources are nonetheless invested even for an event that may or may not happen. Preparedness and resilience are thus promoted as answers to events that can or cannot be prevented from happening. It is through these modes of governing that anticipation, pre-emption, preparedness as protection, and surveillance are used as modes of governance in which resilience and security become entangled and form a “resilience-security-nexus” (Pospisil & Gruber, 2016) to address the “catastrophes to come” (Aradau & van Munster, 2012) in the present.

## Conclusion: Innovation, Tech Fix, and Strategic Ignorance of Security Legacies

The enduring presence of space debris in orbit highlights the long-term consequences of military activities in space. Unlike terrestrial military waste, which can be managed or, to some extent, contained, space debris remains largely uncontrollable; it continuously orbits the Earth at high velocities and increases the risk of collisions with operational satellites and other space infrastructure—collisions that will likely generate even more debris, leading to a self-perpetuating cycle that exacerbates the problem over time. As the material remnants of past military operations, such as anti-satellite (ASAT) tests, missile launches, and the deployment of military satellites during the Cold War, space debris influences both present and future space activities, and poses a persistent threat to each. The legacy of military control over space monitoring systems, such as the U.S. Space Surveillance Network (SSN), means that much of the knowledge about space debris is tied to national security frameworks, which often prioritize secrecy and restrict access to information. This has led to a fragmented and opaque global governance structure in which collaboration and transparency are limited and efforts to develop comprehensive strategies for debris mitigation and management are hindered.

Considering that ESA models suggest that even if launches were halted today, the number of debris parts would rise nonetheless (ESA, 2024, p. 116), the common consensus among experts tends to advocate for more technological innovation, specifically in the domain of Active Debris Removal technology, to engineer a way out of the current environmental predicament (cf. Pelton et al., 2020, p. 280). A temporal shift—to understand space debris as the result of past activities and as an influence on future practices—prompts us to rethink our current innovation paradigm and highlight what is often overlooked: the practices of care and maintenance that keep our space infrastructure operational. Space debris and their related practices of care and maintenance thus provide a window into the limitations of technological progress and the narratives of newness or innovation, which are so prevalent in the space sector.

A focus on the everyday practices of space debris engineers then brings into focus the frictions and obstacles of a global governance system that is dominated by security actors. The current space traffic management system is a patchwork of ad-hoc arrangements; it is troubled by an ever-present imperative of secrecy, which, as we attempted to demonstrate, correlates with a troubling affective atmosphere of uncertainty, distrust, and unpredictability. These issues are unlikely to be resolved by innovation alone if there are not also significant changes in the international legal order concerning outer space. This is particularly true when one considers that technological innovation has been one of the key drivers of the increased usage of orbital space: for the most part, innovation has meant that more things are being launched into space, not less. The hype around satellite-based internet constellations, for example, often glosses over engineers’ struggle to maintain the space infrastructure that already exists, “the heritage that space debris is,” in the face of increasing orbital congestion. Then again, this is not so surprising, as care work often tends to be *made invisible* both institutionally and in public discourse (cf. de la Bellacasa, 2011).

Another issue that pro-innovation policy discourses often omit is the impact that the increased use of space—even with stricter sustainability measures—is likely to have on the upper atmosphere. Increased use of space will likely come with an increased deposit of metals as more and more satellites burn up on reentry, endangering the ozone layer (Ferreira et al., 2024) and potentially changing the atmosphere’s reflective properties (Murphy et al., 2023). Flamm and colleagues (2025) have referred to this lack of attention as “atmosphere-blindness.” Innovation in active debris removal technologies will not be able to solve this issue because they do not address the underlying polluting processes and systems. Only globally agreed-upon limits on the use of space have the potential to curb orbital and atmospheric pollution. As Flamm et al. (2025, p. 3) point out: “[de-orbiting] is not merely a technical, environmental problem to be fixed but also an inherently political matter of planetary-scale environmental justice” (cf. Thiele & Boley, 2023).

Emphasizing care work, often hidden from view but necessary to keep spacecraft safe in an increasingly dangerous space environment, 24 h a day, 365 days a year, reveals the importance of human labor and human creativity in maintaining the infrastructures we rely upon. Approaching techno-scientific care practices in this way allows for a different set of concerns to emerge and complicates the dominant discourse in visions for an intensified usage of space enabled through sheer feats of innovation. Given the distance from, the technical complexity of, and the growing concerns about the issue of space debris, we consider this focus on care and maintenance practices (cf. Tronto, 1998; de la Bellacasa, 2011) an important contribution towards understanding this issue in a more complete and robust manner—to keep things centered around concrete earthly doings but cognizant of the particular challenges that arise from inevitably imbricated security logics.

We see current regulatory efforts such as the *Space Debris Mitigation Guidelines* developed by the Inter-Agency Space Debris Coordination Committee (IADC), as well as the European Space Agency’s *Zero Debris Initiative*, as important steps toward fostering more sustainable space practices. These frameworks signal a growing recognition of the need to mitigate harm and introduce standards for responsible behavior in orbit. However, we are also concerned that, in the current climate of accelerating militarization, space debris is increasingly sidelined (again)—framed as a technical nuisance rather than a pressing political and environmental issue. At the same time, it is exacerbated by the rapid proliferation of satellite launches, mounting geopolitical competition, and the strategic positioning of space infrastructures in preparation for future conflicts. The U.S. Space Force’s growing budget, China’s expanding constellation programs, and the increasing number of dual-use missions across nations all exemplify this trend. The rise of commercial mega-constellations like Starlink and Kuiper—often launched under the banner of connectivity and innovation—further complicate the regulatory landscape, as thousands of satellites are deployed with limited mechanisms for global coordination or accountability.

Just as terrestrial and marine debris reflect our societal values and practices, space debris encapsulates our historical and cultural attention and should prompt us to reflect on both sustainability and responsibility in extraterrestrial environments.

Thus, infrastructure should be understood not only to ground the possibility of hybrid networks of “Planet-Space,” but also in the sense that space debris grounds us—confining us to Earth instead of leaving a polluted Earth behind for other planets. This duality calls for us to understand orbital infrastructures as simultaneously threatened and threatening. The prevailing mindset to exploit the future for present gains disregards the ethical imperative for stewardship and accountability and kicks them down the road to those who will inherit the consequences of our actions. Instead, we should ask ourselves what kind of world we wish to bestow upon future generations and recognize our responsibility in shaping it. The material remains of previous and current space activities are crowding our orbits, they are “imbricated in the management of the future as a material force” (Damjanov, 2017, p. 180), and require more than technological advancement alone. They demand a rethinking of our ethical responsibilities towards our shared space commons.

More broadly, the case of space debris invites us to rethink the ethics of technology not as an abstract exercise in principle-setting, but as a situated and collective practice of care. As we have shown, responding to the growing problem of orbital waste is not just a technical challenge but a deeply ethical one—raising questions about responsibility, temporality, and the limits of innovation. Following Jasanoff’s (2016) call for a more anticipatory and democratic approach to technological governance, we suggest that the issue of space debris exemplifies a broader need to rethink how innovation is framed, justified, and pursued—not only in outer space, but across domains increasingly shaped by rapid technological acceleration and capitalist imperatives (Klinger, 2021; Van de Poel & Verbeek, 2006).

STS scholars have long pointed out that technologies are not neutral tools but embed particular social, political, and economic logics. In the current moment, where innovation is often equated with progress and speed, the ethical imperative to “move fast” can obscure the long-term consequences and uneven risk distributions of new technologies. The space debris crisis reminds us that accumulation—of waste, systems, and techno-futures—has material and moral consequences. As Arthur Schopenhauer (1851) astutely observed, “Any stupid boy can crush a beetle. But all the professors in the world can’t make one.” Similarly, we can carelessly fill orbital space with debris in a matter of decades, but we lack the technology to reverse this damage once done.

Sometimes, doing less—slowing down, resisting the compulsion to innovate for innovation’s sake—might be the more ethical choice. Care, in this sense, means not only attending to the harms already caused, but also cultivating the capacity to say no, to pause, and to imagine forms of responsibility that are not driven by acceleration, but by maintenance, preservation, and restraint.

When we were co-teaching a class on space debris to a group of university students, it was the first time many students had heard of space debris, and their reactions were swift and visceral. There was a sense of disbelief—how could such a massive issue exist beyond the radar of public attention? One student exclaimed, “It’s outrageous—we’ve polluted even space now?” What began as quiet curiosity turned into heated debate: Should we regulate launches more strictly? Could we hold private companies accountable? Why, some students asked, do we seem unable to learn from past mistakes—allowing the damage done to Earth’s environment to now extend into Earth’s orbits? There was a shared sense of sadness in the room, along with a growing urgency. The classroom became a space not only for critique but also for imagining alternatives—a place where technical and sociopolitical perspectives came together, and where care was expressed through listening, questioning, and thinking collectively. Some students proposed bold ideas that go beyond current policy debates—for example, prioritizing environmental concerns over personal convenience, even suggesting we should stop launching space technologies altogether, “even if that means we can’t watch Netflix anymore.” Others proposed more collective approaches: deciding together which technologies are genuinely needed (such as climate-related science missions) and which serve mainly commercial interests (such as mega-constellations for profit).

Encounters like these highlight the importance of fostering ethical reflection and care before crises deepen. They also show the value of open, interdisciplinary dialogue in uncovering what is often hidden by dominant narratives of innovation and security, and in exploring more radical, care-oriented alternatives for the future of space.

## Data Availability

Not applicable.

## Footnotes

1 NewSpace refers to an increasing commercialization and privatization of outer space, which at once spurs enthusiasm and critique. A new popular fascination with colonizing Mars, new legislative practices and regulations empowering private enterprises to exploit outer space, and the channeling of funds to support the space industry have disrupted Cold War era paradigms of national big science projects in outer space, which, until now, have mainly been led by governments.

2 “From the historical perspective, deliberate explosions are essentially extinct as a category, having been employed primarily when a national security payload could not confidently be recovered within the launching country’s borders, as an end-of-mission procedure, or as a design/operational procedural flaw. Deliberate collisions continue on an irregular basis with ASAT tests; note that while higher altitude ASAT tests (SOLWIND, FY-1 C) have—by their longer-term environmental significance—influenced both international guidelines and subsequent tests (USA 19, Microsat-R). Even low-altitude tests can significantly affect the environment” (Anz-Meador et al., 2022, p. 38).

3 The authors of this article have presented joint work at the international, interdisciplinary symposium: The Future of Near-Earth Space. Space Debris and Space Sustainability in Rome, the European Workshops on International Studies (EWIS) in Istanbul and the European Association for the Study of Science and Technology (EASST) in Amsterdam (all 2024). Between 2020 and 2024, two of the authors have co-taught sessions on Space Debris in the Master Program “Science and Technology Studies” (University name will be added after peer review) with students from social science and natural sciences.

4
*Epistemic uncertainties* refer to uncertainties rooted in the limits of what can be known within a given field or practice. As Knorr-Cetina (1999) argues, knowledge production is inherently partial and situated, shaped by the instruments, practices, and epistemic cultures in which it unfolds. In the context of space debris mitigation, such uncertainties arise from limitations in tracking capabilities, incomplete data on debris characteristics, and the difficulty of modeling long-term orbital dynamics. These are not simply technical shortcomings but reflect the open-ended and contingent nature of scientific knowledge in complex sociotechnical systems.

5 The AN/FPS-85 in Florida.

6 Star and Griesemer (1989) introduced the term to describe how actors with different perspectives—such as scientists, policymakers, or engineers—can collaborate without fully agreeing on meanings or goals. In the context of space debris, boundary objects might include orbital risk models, debris catalogs, or mitigation standards, which allow coordination among actors (e.g., engineers, regulators, and international agencies) despite divergent interests, priorities, or understandings of the problem.
